# The Influence of Three Commercial Soy Lecithin-Based Semen Extenders and Two Spermatozoa Concentrations on the Quality of Pre-Freeze and Post-Thaw Ram Epididymal Spermatozoa

**DOI:** 10.3390/ani14081237

**Published:** 2024-04-20

**Authors:** Malam Abulbashar Mujitaba, Gabriella Kútvölgyi, Judit Radnai Szentpáli, Viktória Johanna Debnár, Alexandra Tokár, Nóra Vass, Szilárd Bodó

**Affiliations:** 1 Department of Animal Nutrition and Physiology, Faculty of Agriculture and Food Sciences and Environmental Management, University of Debrecen, Böszörményi Street 138, H-4032 Debrecen, Hungary; malam.abulbashar@agr.unideb.hu (M.A.M.); vassnora@agr.unideb.hu (N.V.); 2 Doctoral School of Animal Science, University of Debrecen, H-4032 Debrecen, Hungary; 3 Department of Precision Livestock Farming and Animal Biotechnics, Institute of Animal Sciences, Hungarian University of Agriculture and Life Sciences, Kaposvár Campus, Guba Sándor Street 40, H-7400 Kaposvár, Hungary; debnar.viktoria.johanna@uni-mate.hu (V.J.D.); bodo.szilard@uni-mate.hu (S.B.); 4 Institute of Horticultural Science, Hungarian University of Agriculture and Life Sciences, Buda Campus, Villányi Street 29-43, H-1118 Budapest, Hungary; szentpaliradnai@gmail.com; 5 Festetics György Doctoral School, Hungarian University of Agriculture and Life Sciences, Deák Ferenc Street 16, H-8360 Keszthely, Hungary; tokar.alexandra@uni-mate.hu

**Keywords:** AndroMed^®^, BioXcell^®^, OviXcell^®^, soy lecithin, ram epididymal spermatozoa, cryopreservation, membrane integrity

## Abstract

**Simple Summary:**

Studies have revealed a rapid global and continental loss of genetic resources for native sheep breeds that is more critical in Europe and the Caucasus region. Therefore, an urgent step is needed to halt this negative trend. Viable and functional epididymal spermatozoa could be retrieved from castrated, slaughtered, or accidentally dead animals with good pregnancy outcomes; however, many factors reported to affect the quality and cryo-tolerance of artificial vagina-collected, as well as electro-ejaculated, ram spermatozoa have not been extensively studied in ram epididymal spermatozoa despite being a cheap alternative for gene conservation. Given the context mentioned above, we assessed the effects of three different commercial soy lecithin-based semen extenders (AndroMed^®^, BioXcell^®^, and OviXcell^®^) and two spermatozoa concentrations (200 × 10^6^/mL vs. 400 × 10^6^/mL) on the freezability of ram epididymal spermatozoa. BioXcell^®^ and OviXcell^®^ produced significantly higher post-thaw specific kinematics and better protected the ram epididymal spermatozoa head membrane compared to AndroMed^®^. In contrast, the normal tail morphology is better maintained in AndroMed^®^. The 400 × 10^6^ spermatozoa/mL concentration better preserved the ram epididymal spermatozoa head membrane integrity. The ideal concentration for cryopreserving ram epididymal spermatozoa is 400 × 10^6^ spermatozoa/mL. However, the extenders must be optimized for more effective ram epididymal spermatozoa freezing.

**Abstract:**

There are limited studies on the factors affecting the success of ram epididymal spermatozoa (REPS) cryopreservation. On this note, the current study assessed the influence of three commercial soy lecithin-based semen extenders, AndroMed^®^ (AND), BioXcell^®^ (BIO), and OviXcell^®^ (OVI), and two concentrations (400 × 10^6^ vs. 200 × 10^6^ spermatozoa/mL) on the pre-freeze and post-thaw quality of REPS. The REPS were retrieved from nine adult rams’ testes and diluted with each of the three extenders to both concentrations. Straws were frozen manually. Standard motility (SMP) and kinematic parameters (KPs) were assessed via a CASA, while spermatozoa viability, morphology, and acrosomal integrity were assessed via the Kovács–Foote staining technique. The concentration did not significantly affect the pre-freeze and post-thaw SMP and KPs of REPS. BIO and OVI had significantly higher pre-freeze and post-thaw BCFs, post-thaw VAP, and the percentage of all intact heads than AND. In contrast, AND had a significantly lower percentage of REPS with tail defects than BIO and OVI. The 400 × 10^6^ spermatozoa/mL concentration resulted in a significantly higher percentage of all intact heads than the 200 × 10^6^ spermatozoa/mL concentration. Freezing significantly increased tail defects and decreased the percentage of REPS with distal cytoplasmic droplets. The cryopreservation of REPS at the 400 × 10^6^ spermatozoa/mL concentration is recommended. All three extenders must be optimized to preserve the viability, membrane integrity, and better normal morphology of REPS; the reason for increased tail abnormality after the freezing/thawing process needs to be studied.

## 1. Introduction

Semen cryopreservation is an important technique that facilitates modern assisted reproductive technologies and ensures the conservation of valuable animal genetic resources (AGRs) in the form of frozen semen for several years or decades, while maintaining its viability and fertilizing ability. Sperm collection in animals with erectile dysfunction is compromised or not feasible via artificial vaginas or electro-ejaculators [1]. Therefore, epididymal spermatozoa (EPS) collection and cryopreservation provides the cheapest and easiest alternative means of collecting spermatozoa to conserve AGRs of a valuable sire in the case of sudden death or castration [2]. Moreover, studies in different species proved that EPS resulted in good-to-excellent pregnancy rates following AI in boar (92.0%) [3], cattle (58.8%) [4], sheep (87.5%, 58.5%, and 55.0%) [5,6,7], goats (61.2%) [8], stallions (27.8% and 64.0%) [9,10], and red deer (75.0%) [11]. Therefore, it is considered the most viable, cheapest, and easiest way to conserve the genetic resources of endangered, threatened, or valuable animals that die accidentally.

Semen extenders are solutions that provide nourishment and protect sperm cells from injury during the cooling and freezing process. They are one factor that affects the fertility of cervical insemination [12]. Some researchers have reported that they greatly influence the quality of frozen–thawed EPS in sheep [12,13] and alpacas [2]. Extenders can be conventional or commercially prepared. The most used ram semen extender is tris-citric egg yolk. The commercially available ones are classified based on their origin/composition. They include those that are soy lecithin-based (AndroMed^®^, BioXcell^®^, Biociphos Plus^®^, Botu-Bov^®^–soy lecithin, and OviXcell^®^), egg-yolk-based (Biladyl^®^, Botu-Bov^®^ Triladyl^®^, and BullXcell^®^), milk-based (INRA96^®^), and protein-free (OptiXcell^®^) [14,15,16,17,18]. Different studies were conducted on the effects of commercially prepared semen extenders on the freezability of the spermatozoa of bulls [15,16,19,20], buffalo [21], goat bucks [22], and rams [23,24,25]. In recent years, there has been a call by researchers against the use of egg-yolk-based extenders due to the wide variability of their components and microbial contamination risk, leading to endotoxin production, reducing spermatozoa post-thaw viability and acrosomal integrity [25,26]. An alternative cold-shock protector for egg yolk is plant-based lecithin. Several studies have been conducted on the effects of different semen extenders on the freezability of artificial vagina (AV)-collected ram spermatozoa [27,28,29]; however, there have been fewer studies on ram epididymal spermatozoa (REPS), particularly on the effects of soy lecithin-based commercially available semen extenders [12,13]. Most of the studies on the effects of soy lecithin-based semen extenders primarily focused on AV-collected ram spermatozoa [17,25,26,30,31,32]. Moreover, the studies conducted on REPS were mostly on the effects of collection methods [13], handling/storage conditions or transportation temperature [33,34,35], washing [36], egg yolk-based extenders [12,13], and the effects of buffers and sugar combinations [37] on their post-thaw quality characteristics. This being the case, there is a need to explore other factors affecting REPS’s post-thaw quality.

The dilution rate or sperm-freezing concentration effect is another exciting factor worth investigating regarding REPS freezability. Some researchers have reported it to affect the quality/success of AV-collected cryopreserved spermatozoa in sheep [26,38,39]. The lower concentration (200 × 10^6^ spermatozoa/mL) was reported to result in better post-thaw quality parameters than the higher doses (400 × 10^6^ or 800 × 10^6^ spermatozoa/mL) [26,40], however, extreme dilution was found to negatively affect the membrane integrity of ram spermatozoa and cause capacitation-like changes [41], and cryopreservation has an additive effect that damages the cells [38]. Moreover, for a successful artificial insemination program, the technique employed in depositing spermatozoa into the receptive female reproductive tract determines the dilution rate [39]. Hence, it is important to identify the most ideal dilution rate/sperm concentration with which to freeze REPS. Moreover, there are fewer or no studies on the ideal spermatozoa concentration of REPS that leads to less detrimental effects on its post-thaw quality. On this note, the current study attempted to investigate the effects of three different commercially available soy lecithin-based semen extenders (AndroMed^®^ (AND), BioXcell^®^ (BIO), and OviXcell^®^ (OVI), with compositions detailed in Table 1) and two different spermatozoa concentrations (400 × 10^6^ vs. 200 × 10^6^ spermatozoa/mL), or their most suitable interactions, on the freezability of REPS. The current study did not consider the breed effect because the sole aim was to identify the ideal concentration, extender, or their most suitable interactions for freezing REPS, regardless of the breed, to enhance the gene conservation of local sheep breeds.

## 2. Materials and Methods

### 2.1. Media, Reagents, and Materials

Three different commercial semen extenders, AndroMed^®^ (AND) (13503/1200 CSS One-step, 200 mL), BioXcell^®^ (BIO) (016218 Easy to use, 250 mL), and OviXcell^®^ (OVI) (020997 Ready-to-use extender, 100 mL), were purchased from Minitube Ltd. (Tiefenbach, Germany) and IMV technologies (L’Aigle, France). The AND and BIO extenders were reconstituted according to the manufacturers’ guidelines, filled into sterilized 10 mL centrifuge tubes, and stored at frozen conditions until required. All other plastic wares were purchased from Falcons^®^ (Corning Inc., Corning, NY, USA), and 0.25 mL transparent semen straws were purchased from IMV Technologies (L’Aigle, France).

### 2.2. Study Location and Testicle Collection

The study was conducted at the Hungarian University of Agriculture and Life Sciences, spermatology laboratory, Herceghalom, Hungary. Nine pairs of intact testes were collected from 9 adult healthy rams (with health status according to the relevant EU regulations) of different breeds, Merino (4), Racka (3), and Dorper (2), from slaughterhouses in Hungary between November 2022 and March 2023. They were transported to the laboratory in a cold box within 2 h and processed individually within 24 h to simulate field conditions, as described by Egerszegi et al. [43].

### 2.3. Epididymal Sperm Collection

**Figure 1:**
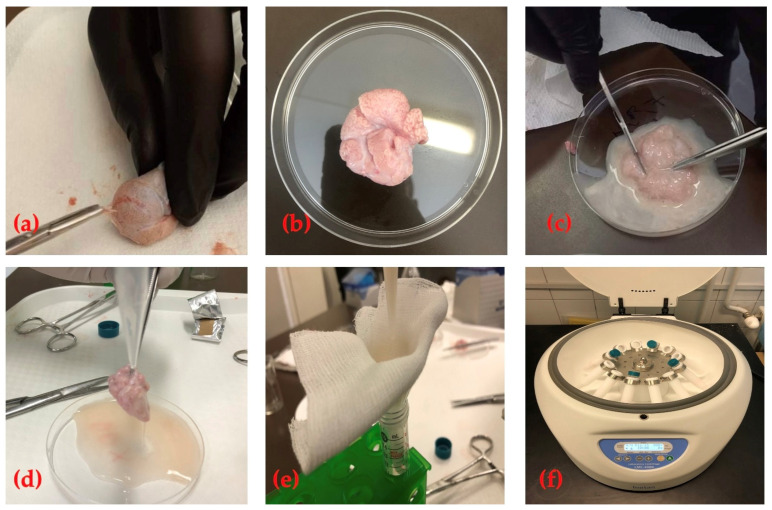
Epididymal sperm collection via the slicing method. (**a**) Stripping the CE for slicing. (**b**) Stripped CE ready for slicing. (**c**) Slicing the stripped CE. (**d**) Rinsing the sliced CE. (**e**) Filtering the retrieved epididymal spermatozoa. (**f**) Centrifuging the retrieved epididymal spermatozoa.

The testes were weighed using a digital weighing scale after removing the scrotal sac and lamina parietalis of the tunica vaginalis. Each cauda epididymis (CE) was carefully separated and weighed, and the spermatozoa were retrieved through slicing. The visceral layer of the tunica vaginalis covering the CE was carefully removed to avoid blood contamination. The stripped CE was washed with a PBS solution and then sliced with a scalpel in a Petri dish containing 3 mL of a tris-citric acid fructose buffer solution (Tris (Hydroxyl methylamino methane), 3.028 g; citric acid monohydrate, 1.70 g; fructose, 1.25 g; and distilled water up to 100 mL), as described by Ahmed et al. [36]. The sliced CE was placed in the tris buffer solution for 10 min to enhance spermatozoa collection, rinsed with 2 mL of the tris buffer, and filtered with gauze sheets; the final volume was then recorded. The tris buffer solution was added to each sample from each CE, making an equal volume of 10 mL, and centrifuged at 880 g for 10 min, according to Ahmed et al. [36]; see Figure 1. Finally, the supernatant was removed, and the pellets that were retrieved from both CEs of the same ram with a good mass motility score of 4–5 were mixed.

### 2.4. Sample Dilution, Equilibration and Freezing

Samples were checked for concentration with a Makler counting chamber (Sefi Medical Instruments, Haifa, Israel), using a phase-contrast microscope at ×200 magnification. Part of the sample was taken and divided into three aliquots, and each of the aliquots was diluted with one of the commercial semen extenders to a concentration of 400 × 10^6^ spermatozoa/mL at room temperature to give AND 400, BIO 400, and OVI 400. Part of each extended sample was aliquoted again and further diluted with the corresponding extender to a final concentration of 200 × 10^6^ spermatozoa/mL, giving AND 200, BIO 200, and OVI 200. The extended samples were manually filled and sealed using polyvinyl alcohol (PVA) into well-labelled and color-coded French Mini straws.

**Figure 2:**
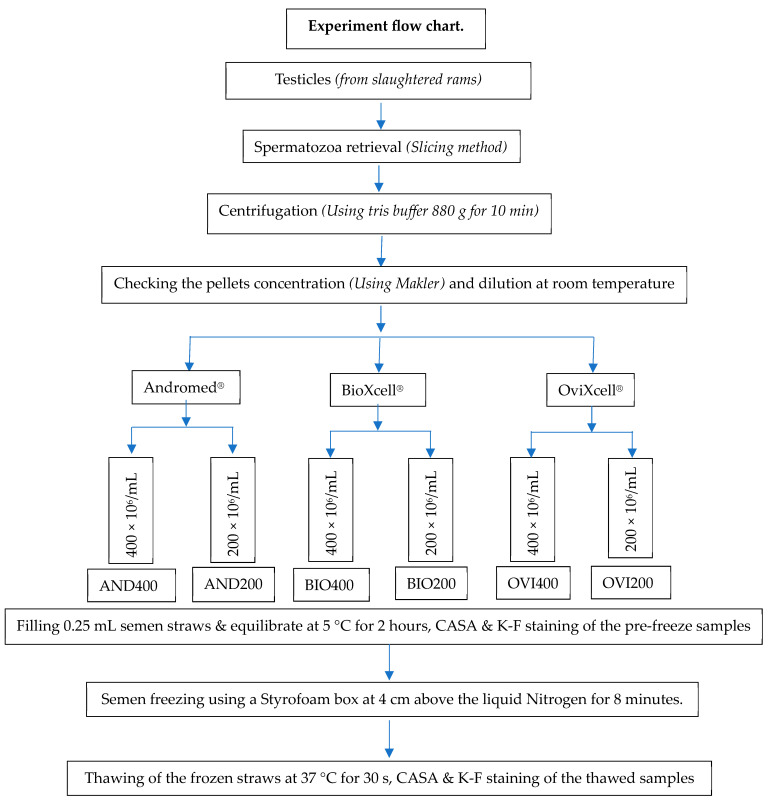
Experiment flowchart.

The filled and sealed straws were equilibrated in a refrigerator (5 °C for 2 h). The freezing of REPS was conducted in a similar way as conventional AV-collected spermatozoa freezing. It was carried out manually in a Styrofoam box at 4 cm above the liquid nitrogen (LN_2_) for 8 min. Finally, the frozen straws were plunged into the LN_2_ for permanent storage. After about 2 weeks, the frozen samples were thawed (37 °C for 30 s) and assessed for standard motility and kinematic parameters. Smears were prepared for membrane integrity and morphology evaluation (Figure 2).

### 2.5. Sample Quality Assessment

#### 2.5.1. Standard Motility and Kinematic Parameters

Pre-freeze and post-thawed spermatozoa’s motility and kinematic parameters were assessed using a computer-assisted sperm analyzer (CASA) (Sperm Vision^TM^ Version 3.8 software, Minitübe Ltd., Tiefenbach, Germany). The samples were diluted to a 50–60 × 10^6^ spermatozoa/mL concentration using the same extender. At least 10 random fields per sample or a total of 500 spermatozoa were analyzed for standard motility (total motility (TM, %) and progressive motility (PM, %)) and kinematic parameters: curvilinear velocity (VCL, μm/s), average path velocity (VAP, μm/s), straight line velocity (VSL μm/s), linearity (LIN = VSL/VCL × 100, %), straightness (STR = VSL/VAP × 100, %), beat cross frequency (BCF, Hz), wobble (WOB = VAP/VCL × 100, %), and amplitude of lateral head displacement (ALH, μm), as described by Goovaerts et al. [44], Kang et al. [4], and Bergstein-Galan et al. [45].

#### 2.5.2. Viability and Morphology Assessment

**Figure 3:**
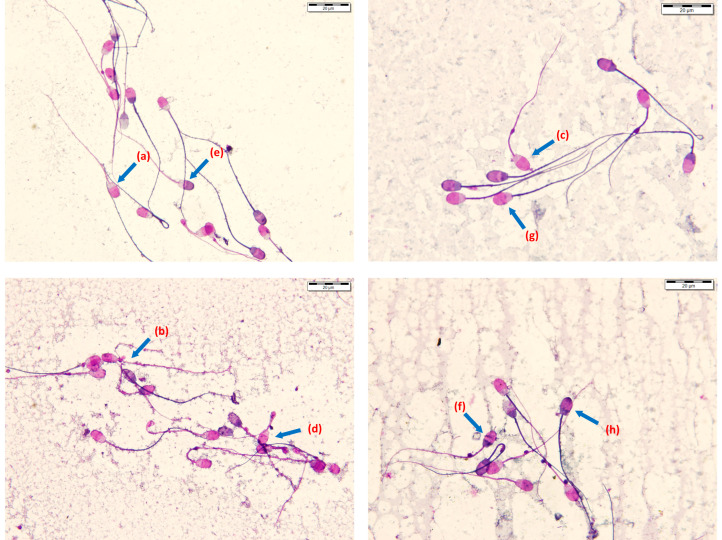
The different post-thaw ram epididymal spermatozoa categories stained with the modified Kovács–Foote staining technique (magnification × 1000, using a light microscope with an oil immersion objective). (**a**) Intact head, intact tail, and acrosome membrane (Intact: IHITIA). (**b**) Intact with a proximal droplet (IPD). (**c**) Intact with distal droplet (IDD). (**d**) Intact with a bent tail (IBT). (**e**) Intact head, tail, damaged acrosome (IHITDA). (**f**) Damaged head with intact tail (DHIT). (**g**) Intact head with damaged tail (IHDT). (**h**) Damaged head, damaged tail, and damaged acrosome (DHDTDA).

The acrosome, head, and tail membrane integrity, as well as the morphology, of spermatozoa were evaluated via a modified Kovács–Foote staining method, using a 0.16% Chicago sky blue 6B (Sigma-Aldrich, St. Louis, MO, USA, C-8679) viability stain, neutral red (Sigma N 2880), formaldehyde fixation, and 7.5% Giemsa solution (Sigma GS-500) in distilled water prepared freshly before use for acrosome staining [46,47]. The procedure involved the viability staining of the diluted samples and the air-drying of the slides, fixation for 4 min, followed by rinsing with tap and distilled water, and finally staining with a Giemsa solution for 3.5–4 h. After this, rinsing with tap water and the differentiation of the stained slides in distilled water for 2 min were carried out for better categorization of the spermatozoa. Slides were evaluated using an oil-immersion objective with bright-field microscopy at ×1000 magnification with a yellow filter for better live/dead differentiation [46]. A total of three hundred cells were counted on each slide and classified into eight categories: intact head, intact tail, and acrosome membrane (Intact); normal morphology (IHITIA); intact with a proximal cytoplasmic droplet (IPD); intact with a distal cytoplasmic droplet (IDD); intact with a tail defect (bent, broken, hairpin curved, or coiled tail) (IBT); intact head and tail, damaged acrosome (IHITDA); damaged head with intact tail (DHIT); intact head with damaged tail (IHDT); and damaged head, damaged tail, and damaged acrosome (DHDTDA), as described by Kútvölgyi et al. [46]. Different spermatozoa categories are shown in Figure 3. In addition, all distal cytoplasmic droplets and all bent, hairpin-curved tails were counted regardless of intact or damaged membranes, and per cent, all intact spermatozoa (IHITIA + IPD + IDD + IBT), all intact heads (IHITIA + IPD + IDD + IBT + IHITDA + IHDT), and all intact tails (IHITIA + IPD + IDD + IBT + IHITDA + DHIT) were also calculated. The values obtained for each category were presented in percentages.

### 2.6. Data Analysis

Data from pre-freeze, post-thaw, and Kovács–Foote-stained REPS were collected, recorded, and analyzed for descriptive statistics, using IBM^®^ SPSS^®^ statistical software version 29. Normality was checked using a Shapiro–Wilk test, and transformations were achieved using a two-step transformation. A general linear model using two-way analysis of variance was used to analyze the effects of extender and sperm concentration (400 *×* 10^6^ vs. 200 *×* 10^6^ spermatozoa/mL), as well as their interaction, on standard motility, kinematic parameters, viability, and morphological parameters, with the level of the significance set at *p* < 0.05. Means were separated using the Tukey post hoc test. The effects of freezing using different commercial soy lecithin-based semen extenders and the overall effects of freezing and thawing on the percentage of distal droplets and tail defects were analyzed using Student’s paired-sample *t*-test, and the significance difference was checked using a two-tailed test. The results are presented as means ± standard errors of means (SEs). 

## 3. Results

### 3.1. General Parameters of Ram Epididymal Spermatozoa

In the current study, we determined certain parameters related to the ram testicles and cauda epididymal (CE) weight, in addition to the concentration of the spermatozoa retrieved from rams of different breeds (Table 2). The mean testicular weight, epididymal weight, and spermatozoa concentration obtained were 157.78 ± 22.15 g, 14.25 ± 1.38 g, and 9061.44 ± 845.53 × 10^6^/mL, respectively.

### 3.2. Effects of Three Different Commercial Soy Lecithin-Based Semen Extenders and Two Spermatozoa Concentrations on the Standard Motility and Kinematic Parameters of Pre-Freeze Ram Epididymal Spermatozoa

The effects of the three different commercial soy lecithin-based semen extenders and two spermatozoa concentrations on pre-freeze REPS are presented in Table 3. There was no significant (*p* > 0.05) interaction between the extender and the spermatozoa concentrations for all the parameters studied, so we present the main treatment effect. Similarly, the standard motility and all kinematic parameters showed no significant (*p* > 0.05) difference among the extenders and between the two spermatozoa concentrations, except for BCF. The BIO and OVI extenders had significantly (*p* < 0.05) higher BCFs (30.18 ± 1.1 and 29.99 ± 1.0 Hz) than the AND extender (26.80 ± 0.8 Hz).

### 3.3. Effects of Three Different Commercial Soy Lecithin-Based Extenders and Two Spermatozoa Concentrations on Standard Motility and Kinematic Parameters of Post-Thaw Ram Epididymal Spermatozoa

Table 4 presents the effects of the three commercial soy lecithin-based semen extenders and two spermatozoa concentrations on the REPS’s post-thaw standard motility and kinematic parameters. There was no significant (*p* > 0.05) interaction between the extender and spermatozoa concentrations for all of the studied parameters, so we present the main effect of the extenders and the spermatozoa concentrations. The standard motility parameters of the post-thaw REPS were also not significantly (*p* > 0.05) different among the extenders and between the spermatozoa concentrations. The BIO and OVI extenders had statistically the same post-thaw VAPs (77.78 ± 3.2 vs. 80.48 ± 3.1 μm/s) and BCFs (32.81 ± 1.1 vs. 32.46 ± 1.0 Hz) and were significantly (*p* < 0.05) higher than the AND extender (67.72 ± 3.5 μm/s and 28.72 ± 0.9 Hz). Moreover, OVI had significantly higher (*p* < 0.05) per cent WOB than the AND extender (50.56 ± 0.8 vs. 47.67 ± 0.7 %), while BIO and OVI were statistically the same (49.56 ± 0.9 vs. 50.56 ± 0.8 %). All other kinematic parameters were statistically the same (*p* > 0.05) among the extenders and between the spermatozoa concentrations.

### 3.4. Effects of Different Soy Lecithin-Based Commercial Semen Extenders and the Two Spermatozoa Concentrations on the Post-Thaw Viability and Morphological Characteristics of Ram Epididymal Spermatozoa

The effects of different soy lecithin-based commercial semen extenders and the two spermatozoa concentrations on the post-thaw viability and morphological characteristics of the REPS are presented in Table 5. There was no significant (*p* > 0.05) interaction between the extenders and the spermatozoa concentrations. Similarly, neither the extender nor the spermatozoa concentration significantly affects the percentage of the post-thaw REPS with IHITIA. The AND extender had a significantly (*p* < 0.05) lower percentage of the intact REPS with bent tails (IBT), all intact heads, and all bent tails categories (2.56 ± 0.6, 34.64 ± 3.2, and 9.74 ± 1.4%) than the BIO (8.14 ± 1.5, 45.33 ± 3.3, and 18.33 ± 2.4%) and OVI (7.19 ± 1.3, 44.68 ± 2.9, and 17.39 ± 1.7%) extenders. In contrast, the BIO and OVI extenders were statistically the same and had a lower percentage of categories of REPS with DHIT than the AND extender: 2.91 ± 0.7 and 2.53 ± 0.4 vs. 6.31 ± 1.1, respectively. The 400 × 10^6^ spermatozoa/mL concentration resulted in a significantly (*p* < 0.05) higher percentage of all intact head categories than the 200 × 10^6^ spermatozoa/mL (45.15 ± 5.1 vs. 37.95 ± 3.4%) concentration. The extenders and the spermatozoa concentrations did not affect all of the other parameters.

### 3.5. Effect of Freezing with Different Commercial Soy Lecithin-Based Semen Extenders on All Distal Droplets and Tail Defects of Ram Epididymal Spermatozoa

Table 6 presents the effects of freezing REPS with different commercial semen extenders on all distal droplets and tail defects of REPS. Considering that the 200 and 400 million spermatozoa/mL concentrations were statistically the same (Table 5), the data were pooled to assess the effects of freezing REPS on all distal droplets and all bent tails, in addition to the overall effects of freezing. Significant (*p* < 0.05) differences existed between the pre-freeze and the post-thaw distal droplets, as well as the bent tails, in all of the extenders and the overall means: AND, 38.51 ± 4.8 vs. 28.17 ± 2.9% and 5.52 ± 1.3 vs. 9.74 ± 1.4%; BIO, 32.92 ± 5.5 vs. 21.72 ± 2.8 and 11.24 ± 2.7 vs. 18.33 ± 2.4%; and OVI, 26.62 ± 3.6 vs. 20.33 ± 2.5% and 11.31 ± 2.4 vs. 17.39 ± 1.7%. And the overall means were 32.69 ± 2.7 vs. 23.41 ± 1.6 % and 9.29 ± 1.3 vs. 15.15 ± 1.2% for all distal droplets and all bent tails, respectively.

## 4. Discussion

It is well established that cryopreservation decreases spermatozoa viability, functionality, and fertilizing ability [19,39,48]. Furthermore, many factors affecting the success of REPS’s cryopreservation have not been extensively studied, as in AV- and EE-collected ram spermatozoa [26,49]. Among these are the spermatozoa concentration and the diluents used, in particular the readily available commercial soy lecithin-based extenders. On this note, the current study attempted to investigate the earlier-mentioned factors of the pre-freeze quality and freezability of postmortem REPS. Furthermore, the diluents/extenders were reported to affect the freezability of EPS in different species [2,12,13]. Moreover, the animal-based semen extenders were reported to contain variable compositions with a high microbial contamination risk, reducing spermatozoa’s post-thaw viability and acrosome integrity compared to the plant-based extenders [27]. Hence, it is important to identify the ideal commercially available soy lecithin-based diluent and spermatozoa concentration for freezing REPS.

The average weight of the testes and the CE processed in this study (157.78 ± 22.15 and 14.25 ± 1.38 g) were slightly lower than what was reported by Kaabi et al. [34]: 191.11 ± 4.9 and 18.14 ± 0.4 (g), respectively. However, our results presented higher values of standard error, which might be attributed to individual animal differences due to age, season, and breed effects.

The kinematics are important in determining spermatozoa functionality and freezing/thawing success, and spermatozoa with higher BCFs and lower ALH result in a high PM [50]. Similarly, the VAP parameter is preferred over the PM in predicting fresh and post-thaw bull spermatozoa fertilizing potentials [51]. Moreover, the kinematic parameters show relatively high breed similarities in sheep; however, specific kinematic parameters, like the VCL, might vary even between individual sperm from 50 to 320 μm/s in a single field of analysis, and the spermatozoa sub-population with the highest velocity has higher cervical mucus penetration and fertilization rates [50], with the VCL and VAP being the only kinematic parameters that showed a significant positive correlation with cervical mucus penetration in sheep [52] and litter size in pigs [53]. The pregnancy rate in sheep has a strong and significant positive correlation with the spermatozoa PM and VAP (r = 0.62), LIN (r = 0.86), and STR (r = 0.55), but it is negatively correlated with the VCL (r = −0.65), while the average litter size is positively correlated with LIN (r = 0.87) and STR (r = 0.77) [54]. 

The current study demonstrates that there was no significant (*p* > 0.05) difference among the three commercial soy lecithin-based semen extenders, AND, BIO, and OVI, and between the spermatozoa concentration, 200 × 10^6^/mL or 400 × 10^6^/mL, on standard motility parameters of pre-freeze and post-thaw REPS. These findings contrast with the findings of D’Alessandro et al. [39], who reported that the freezing concentration effect on the freezability of AV-collected ram spermatozoa was due to extender differences (milk-based vs. egg yolk-based); however, in the current study, all of the extenders compared were soy lecithin-based, and this might be why we could not observe any significant difference among them. Similarly, Abdussamad et al. [15] reported no significant (*p* > 0.05) difference in the post-thaw TM between two different egg yolk-based extenders in bull cryopreserved spermatozoa and between two soy lecithin-based semen extenders. Our result for the post-thaw motility parameters agrees with Braga et al. [19] and Ondřej et al. [55], who reported no significant difference in motility between the AND and BIO extenders in bull AV-collected post-thaw spermatozoa. It also tallies with that of Akçay et al. [26] in rams. Similarly, Fernandes et al. [27] reported no significant difference (*p* > 0.05) in the post-thaw TM (33.7 vs. 41.7%) and PM (4.6 vs. 5.0%) between the AND and OVI extenders in Portuguese Merino breed AV-collected spermatozoa.

For the pre-freeze kinematics, the BCF was the only parameter that was significantly (*p* < 0.05) different among the extenders. The REPS diluted with the BIO and OVI extenders had significantly (*p* < 0.05) higher BCFs than those in the AND extender. A higher BCF value was reported to be associated with increased fertilization rates [44].

The REPS frozen in the BIO and OVI extenders had statistically the same post-thaw VAP and BCF and were significantly higher (*p* < 0.05) than the AND extender. Therefore, freezing REPS in the BIO and OVI extenders might lead to a higher fertilization rate than that when using the AND extender. This is because the higher BCFs and lower ALH of sperm heads could facilitate zona pellucida penetration [44], and a higher VAP might lead to higher cervical mucus penetration and fertilization rates [52]. The WOB parameter depicts the degree of oscillation of the sperm head/balancing [56]. The spermatozoa concentration did not affect the parameter but differed significantly between the AND and OVI extenders. Moreover, spermatozoa with higher progression tend to have higher cryo-survival and fertilization potentials [57]. Our results of the effects of semen extenders on the WOB parameter contradict the findings of Dorado et al. [58] in goat bucks and Domingo et al. [59] in rabbits, that being that semen extenders have no significant effect on the WOB parameter. With regard to the spermatozoa concentrations, our result was not in agreement with that of Akçay et al. [26] and Nascimento et al. [40], who reported better post-thaw quality parameters in AV-collected ram spermatozoa frozen at 200 × 10^6^/mL than at 400 × 10^6^/mL. This might be due to the differences in spermatozoa source, as well as the extenders’ compositions. Moreover, D’Alessandro et al. [39] and Akçay et al. [26] reported that increasing the freezing concentration to 800 *×* 10^6^/mL has a more significant negative influence on the post-thaw quality of ram spermatozoa.

In the current study, we used the Kovács–Foote viability staining technique to evaluate the REPS’s head, tail, and acrosome membrane integrity, as well as morphology. Although the technique is a subjective evaluation, it is economical, as it does not require a costly device and permits the investigator to see damage/abnormalities in the spermatozoa. Using this method, the acrosome, head, and tail membranes of the sperm can be assessed separately, ensuring the precise determination of lesions’ locations. The retained cytoplasmic droplets are caused by incomplete maturation in the epididymis, leading to abnormal spermatozoa morphology and, thus, impairing viability and capacitation in boars [60,61]. The percentage of distal droplets was also reported to increase significantly with bulls’ age [62], and it is positively correlated with ROS production in men [63]. Additionally, the presence of distal droplets has been associated with a higher percentage of ubiquitinated protein and morphological abnormality, and they also harbored 15-lipoxygenases, which are responsible for mitochondria degradation in ejaculated boar spermatozoa [64,65]; however, they are a normal organelle in EPS, and the complete absence of them indicates spermatogenesis abnormality [66]. Similarly, maintaining tail/flagella integrity is very important because it aids spermatozoa’s heads in achieving fertilization [50]. AND preserved the REPS’s normal tail morphology better than the BIO and OVI extenders did. The 400 × 10^6^ spermatozoa/mL concentration was superior in preserving the REPS’s head membrane integrity compared to the 200 × 10^6^ spermatozoa/mL (45.15 ± 5.1 vs. 37.95 ± 3.4%) concentration. Freezing REPS with the BIO or OVI extender better maintained the REPS’s head membrane compared to the AND extender, as indicated by their significantly higher percentage of all intact head values. The highest value of “all intact heads” that we observed in the current study was in BIO, 45.33 ± 3.3%, and was slightly below what was reported by [36], 51.38 ± 4.44%, using the eosin–nigrosin staining technique. The percentage of spermatozoa with IHDT recorded in the current study (15.52 ± 1.8 to 20.27 ± 2.5%) was similar to that reported in bulls, 20%; boars and rams, 5 to 25% [67]; deer, 20% [68]; and stallions, 19.0% [69].

We supposed that the percentage of “all intact” cells corresponds to the percentage of “live” spermatozoa with intact cell membranes and presumably actively moving spermatozoa, while cells with damaged tails and intact heads are supposed to not move and, hence, be non-fertile in vivo [67]. The percentage of all intact spermatozoa observed in the current study ranges between 19.08 ± 2.2 and 25.03 ± 1.5%. It agrees with Salamon and Maxwell’s report [49] that only about 20–30% of post-thawed ram spermatozoa remain biologically intact.

The highest post-thaw percentage of all distal cytoplasmic droplets observed in the current study (AND: 28.17 ± 2.9%) was comparable to that reported in goat bucks (27.8%) [70], but it was lower than that of Kaabi et al. [34] (55.1 ± 5.3%) in rams under similar conditions; however, in the latter experiment, both proximal and distal cytoplasmic droplets were counted. They retrieved spermatozoa in different ways: no extender was used in the slicing procedure to allow sperm to swim out, and there was no centrifugation step in the protocol. These steps probably enhanced the drifting of the distal droplets in some of the cells. Centrifugation was reported to reduce the number of distal cytoplasmic droplets in collared peccaries (*Pecari tajacu Linnaeus*) [71] and in cat epididymal spermatozoa [72]. Therefore, more studies are needed to confirm this speculation in rams. Freezing REPS with all of the extenders showed a significant difference in the percentage of all distal droplets and all tail defects. We observed that the bent tails increased by about the same percentage as the distal droplets decreased in the frozen samples compared to the pre-freeze condition. The reason for this may be that spermatozoa’s moving tails suddenly get stuck and enclose the droplet and become a spermatozoon with a distal midpiece reflex (also called a hairpin-curved tail), or the osmotic changes during the freezing/thawing process can cause the bending of the tail for some of the distal droplet-bearing sperm. This phenomenon seems to have occurred more in the BIO and OVI extenders than the AND extender, which resulted in a higher percentage of spermatozoa with bent tails in the former extenders than in the latter. Similarly, the overall mean proved that freezing in general significantly (*p* < 0.05) increases the percentage of REPS with tail defects (9.29 ± 1.3 vs. 15.15 ± 1.2%), with a significant decrease in the percentage of all distal droplets (32.69 ± 2.7 vs. 23.41 ± 1.6%). Our result of the percentage of distal droplets was consistent with the findings of Kaabi et al. [34].

## 5. Conclusions

Ram epididymal spermatozoa can behave differently than ejaculated spermatozoa during the freezing/thawing process; the membrane structure could be more unstable, so improving and optimizing the freezing technique of REPS is needed. The BIO and OVI extenders showed significantly higher post-thaw VAP and BCFs and were superior to the AND extender in preserving the ram epididymal spermatozoa head membrane integrity. In contrast, the AND extender was superior in maintaining the normal tail morphology of ram epididymal spermatozoa compared to the BIO and OVI extenders. Freezing significantly decreased the percentage of spermatozoa with distal cytoplasmic droplets and increased the percentage of ram epididymal spermatozoa with tail defects; these phenomena could be connected. All three commercial soy lecithin-based extenders must be optimized to better preserve the viability, membrane integrity, and normal morphology of REPS. Future studies should investigate the effect of extenders and spermatozoa concentration on REPS’s mitochondrial membrane potentials, ATP content, and in vivo fertility. Ram epididymal spermatozoa are suggested to freeze in the 400 × 10^6^ spermatozoa/mL concentration, as it better preserves the head membrane integrity of REPS compared to cryopreserving in the 200 × 10^6^ spermatozoa/mL concentration. The effect of centrifugation on REPS distal cytoplasmic droplets and the reason for increased tail abnormalities after the freezing/thawing process need to be studied.

For postmortem gamete extraction and cryopreservation, selecting the best cryopreservation procedure for ex situ in vitro gene conservation is essential. Using the appropriate extender allows the samples to be stored more successfully; therefore, our research is a valuable step in this regard.

## Figures and Tables

**Table 1:** Compositions of the three commercial soy lecithin-based semen extenders.

AndroMed^®^ (100 mL)	BioXcell^®^ (1000 mL)	OviXcell^®^ (100 mL)
Phospholipids	Glycine (0.2 g/L)	Amino acid
TRIS	TRIS (2.3 g/L)	Buffers
Citric acid	Monohydrate citric acid (2.5 g/L)	
	Sodium citrate (6.2 g/L) Potassium chloride (0.8 g/L) Hydrate of calcium lactate (0.7 g/L)	Salts
Sugars	Fructose (1.2 g/L) Monohydrate lactose (0.8 g/L) Anhydrous glucose (0.5 g/L)	Sugars
Antioxidants	Taurine (0.005 g/L)	Taurine
Glycerol (6.7%)	Glycerol (7.0%/40.2 g/L)	Glycerol
Tylosin (5.7 mg)	Tylosin tartrate (0.33 g/L)	Tylosin tartrate
Gentamicin (28.6 mg)	Gentamycin sulphate (0.24 g/L)	Gentamicin
Spectinomycin (34.3 mg)	Spectinomycin	Spectinomycin sulfate (<0.2%)
Lincomycin (17.2 mg)	Licospectin 100 (0.385 g/L)	Lincomycin hydrochloride
Soy lecithin	Soy lecithin (1.5 g/L)	Soy lecithin
Ultrapure water	Ultrapure water (ad 1000 mL)	Ultrapure water

Sources: Extenders’ leaflets; [42].

**Table 2:** General parameters of the ram epididymal spermatozoa retrieved from different ram breeds.

Parameters	Range	Mean ± SE
Testicular weight (g)	113.07–308.09	157.78 ± 22.15
Cauda epididymal weight (g)	7.89–20.39	14.25 ± 1.38
Spermatozoa concentration (10^6^/mL)	5800–14,240	9061.44 ± 845.53

SE: standard error of means, n = 9.

**Table 3:** Effects of three different commercial soy lecithin-based semen extenders and two spermatozoa concentrations on the standard motility and kinematic parameters of pre-freeze ram epididymal spermatozoa.

Extenders	Standard Motility and Kinematic Parameters									
	TM (%)	PM (%)	VCL (μm/s)	VAP (μm/s)	VSL (μm/s)	LIN (%)	STR (%)	BCF (Hz)	WOB (%)	ALH (μm)
AndroMed^®^	72.22 ± 3.2	64.89 ± 3.4	163.94 ± 5.8	76.85 ± 2.3	54.05 ± 2.4	32.83 ± 1.3	70.00 ± 2.2	26.80 ± 0.8 ^a^	46.61 ± 0.5	5.55 ± 0.2
BioXcell^®^	69.00 ± 3.8	62.44 ± 4.0	168.11 ± 3.9	82.21 ± 2.3	60.64 ± 3.1	35.50 ± 1.6	72.83 ± 2.4	30.18 ± 1.1 ^b^	48.44 ± 0.6	5.21 ± 0.2
OviXcell^®^	67.61 ± 3.7	60.78 ± 3.9	169.06 ± 3.2	83.16 ± 2.2	62.00 ± 3.4	35.94 ± 1.5	73.22 ± 2.2	29.99 ± 1.0 ^b^	48.56 ± 0.7	5.27 ± 0.1
*p*-value	0.633	0.727	0.863	0.336	0.215	0.267	0.463	0.020	0.080	0.695
Conc. (10^6^/mL)										
200	67.85 ± 3.3	61.26 ± 3.4	167.48 ± 3.7	81.14 ± 1.9	59.80 ± 2.5	35.26 ± 1.2	72.85 ± 1.9	29.24 ± 0.9	48.04 ± 0.6	5.25 ± 0.2
400	71.37 ± 2.5	64.15 ± 2.8	166.59 ± 3.6	80.34 ± 1.9	58.00 ± 2.5	34.56 ± 1.2	71.19 ± 1.9	28.71 ± 0.8	47.70 ± 0.5	5.42 ± 0.2
*p*-value	0.170	0.231	0.556	0.379	0.302	0.808	0.584	0.834	0.985	0.181
*p*-value Ext. * Conc.	0.619	0.643	0.852	0.744	0.659	0.887	0.840	0.854	0.946	0.712

Conc.: concentration. TM: total motility. PM: progressive motility. VCL: curvilinear velocity. VAP: average pathway velocity. VSL: straight line velocity. LIN: linearity of movement. STR: straightness. BCF: beat cross frequency. WOB: wobble. ALH: amplitude of the lateral head displacement. Ext. * Conc.: extender * concentration interaction effects. SE: standard error of means, n = 9. Means in the same column with different superscripts ^a,b^ differ significantly.

**Table 4:** Effects of the three different commercial soy lecithin-based semen extenders and two spermatozoa concentrations on the standard motility and kinematic parameters of post-thaw ram epididymal spermatozoa.

Extenders	Standard Motility and Kinematic Parameters (Mean ± SE)									
	TM (%)	PM (%)	VCL (μm/s)	VAP (μm/s)	VSL (μm/s)	LIN (%)	STR (%)	BCF (Hz)	WOB (%)	ALH (μm)
AndroMed^®^	34.89 ± 3.9	27.11 ± 3.4	139.55 ± 6.3	67.72 ± 3.5 ^a^	50.58 ± 3.3	35.72 ± 1.4	74.06 ± 2.3	28.72 ± 0.9 ^a^	47.67 ± 0.7 ^a^	4.41 ± 0.2
BioXcell^®^	38.83 ± 3.5	31.50 ± 3.1	156.72 ± 5.0	77.78 ± 3.2 ^b^	58.96 ± 3.9	37.11 ± 1.8	74.28 ± 2.5	32.81 ± 1.1 ^b^	49.56 ± 0.9 ^ab^	4.42 ± 0.2
OviXcell^®^	37.61 ± 3.7	31.56 ± 3.5	157.39 ± 5.4	80.48 ± 3.1 ^b^	61.46 ± 3.9	38.33 ± 1.7	75.00 ± 2.4	32.46 ± 1.0 ^b^	50.56 ± 0.8 ^b^	4.55 ± 0.2
*p*-value	0.893	0.509	0.191	0.024	0.154	0.554	0.816	0.012	0.044	0.849
Concentrations (10^6^/mL)										
200	34.33 ± 2.3	27.33 ± 2.2	150.40 ± 5.3	75.43 ± 3.2	57.92 ± 3.4	37.74 ± 1.4	75.41 ± 1.9	31.83 ± 0.9	49.37 ± 0.8	4.33 ± 0.1
400	39.89 ± 3.5	32.78 ± 3.1	152.04 ± 4.3	75.22 ± 2.4	56.07 ± 2.8	36.37 ± 1.3	73.48 ± 1.9	30.83 ± 0.8	49.15 ± 0.6	4.58 ± 0.2
*p*-value	0.170	0.249	0.878	0.957	0.534	0.486	0.566	0.400	0.815	0.250
*p*-value Ext. * Conc.	0.723	0.946	0.648	0.855	0.913	0.976	0.959	0.827	0.882	0.927

TM: total motility. PM: progressive motility. VCL: curvilinear velocity. VAP: average pathway velocity. VSL: straight line velocity. LIN: linearity of movement. STR: straightness. BCF: beat cross frequency. WOB: wobble. ALH: amplitude of the lateral head displacement. Ext. * Conc.: extender * concentration interaction effects. SE: Standard error of means, n = 9. Means in the same column with different superscripts ^a,b^ differ significantly.

**Table 5:** Effects of the three different commercial soy lecithin-based semen extenders and two spermatozoa concentrations on the viability and morphological parameters of post-thaw ram epididymal spermatozoa.

Extenders	Viability and Morphological Parameters (Mean ± SE)												
	IHITIA (%)	IPD (%)	IDD (%)	IBT (%)	IHITDA (%)	DHIT (%)	IHDT (%)	DHDTDA (%)	All Intact (%)	All Intact Head (%)	All Intact Tail (%)	All Distal Droplets (%)	All Bent Tails (%)
AndroMed^®^	5.92 ± 1.2	0.87 ± 0.3	9.72 ± 1.4	2.56 ± 0.6 ^a^	0.04 ± 0.0	6.31 ± 1.1	15.52 ± 1.8	59.06 ± 3.4	19.08 ± 2.2	34.64 ± 3.2 ^a^	25.42 ± 2.9	28.44 ± 2.9	9.74 ± 1.4 ^a^
BioXcell^®^	6.55 ± 1.1	0.91 ± 0.2	9.44 ± 1.4	8.14 ± 1.5 ^b^	0.03 ± 0.0	2.91 ± 0.7	20.27 ± 2.5	51.73 ± 3.3	25.03 ± 1.5	45.33 ± 3.3 ^b^	27.97 ± 1.9	21.89 ± 2.7	18.33 ± 2.4 ^b^
OviXcell^®^	7.46 ± 1.3	0.68 ± 0.2	9.33 ± 1.5	7.19 ± 1.3 ^b^	0.02 ± 0.0	2.53 ± 0.4	20.00 ± 1.8	52.79 ± 3.0	24.66 ± 2.4	44.68 ± 2.9 ^b^	27.21 ± 2.6	20.33 ± 2.4	17.39 ± 1.7 ^b^
*p*-value	0.717	0.613	0.981	0.003	0.866	0.155	0.143	0.242	0.094	0.030	0.771	0.100	0.0001
Con. (10^6^/mL)													
200	5.43 ± 1.3	0.70 ± 0.3	9.03 ± 2.1	6.39 ± 1.9	0.04 ± 0.0	4.83 ± 0.8	16.36 ± 2.2	57.23 ± 4.1	21.55 ± 2.8	37.95 ± 3.4 ^A^	26.41 ± 3.7	24.15 ± 4.1	16.81 ± 2.9
400	7.86 ± 1.9	0.94 ± 0.3	9.96 ± 2.1	5.53 ± 1.1	0.02 ± 0.0	3.00 ± 0.9	20.83 ± 4.0	51.83 ± 5.1	24.29 ± 2.9	45.15 ± 5.1 ^B^	27.32 ± 3.4	22.96 ± 3.7	13.49 ± 2.1
*p*-value	0.186	0.079	0.587	0.820	0.703	0.165	0.151	0.160	0.267	0.049	0.760	0.713	0.118
*p*-value Ext. * Conc.	0.337	0.692	0.946	0.819	0.374	0.876	0.783	0.918	0.750	0.724	0.984	0.833	0.277

IHITIA: intact head, intact tail, and acrosome membrane (Intact). IPD: intact with proximal droplet. IDD: intact with distal droplets. IBT: intact with a bent tail. IHITDA: intact head and tail, damaged acrosome. DHIT: damaged head with intact tail. IHDT: intact head with damaged tail. DHDTDA: damaged head, damaged tail, and damaged acrosome. Ext. * Conc.: extender * concentration interaction effect. SE: standard error of the means. Stained with a modified Kovács–Foote staining technique, three hundred cells were evaluated and categorized per slide, using a bright-field microscope with an oil-immersion objective at ×1000 magnification, n = 9. Means in the same column with different superscripts among the extenders ^a,b^ and between the spermatozoa concentrations ^A,B^ differ significantly.

**Table 6:** Effects of freezing and thawing with different commercial soy lecithin-based semen extenders on distal droplets and tail defects of ram epididymal spermatozoa.

	All Distal Droplets		*p*-Values	All Bent Tails		*p*-Values
Extender	Pre-Freeze	Post-Thaw		Pre-Freeze	Post-Thaw	
AND	38.51 ± 4.8 ^a^	28.17 ± 2.9 ^b^	0.002	5.52 ± 1.3 ^a^	9.74 ± 1.4 ^b^	0.003
BIO	32.92 ± 5.5 ^a^	21.72 ± 2.8 ^b^	0.009	11.24 ± 2.7 ^a^	18.33 ± 2.4 ^b^	0.003
OVI	26.62 ± 3.6 ^a^	20.33 ± 2.5 ^b^	0.032	11.31 ± 2.4 ^a^	17.39 ± 1.7 ^b^	0.002
Overall	32.69 ± 2.7 ^a^	23.41 ± 1.6 ^b^	0.0001	9.29 ± 1.3 ^a^	15.15 ± 1.2 ^b^	0.0001

AND: AndroMed^®^ extender. BIO: BioXcell^®^ extender. OVI: OviXcell^®^ extender. SE: standard error of the means, n = 9. Stained with a modified Kovács–Foote staining technique, three hundred cells were evaluated and categorized per slide using a bright-field microscope with an oil-immersion objective at ×1000 magnification. Means in the same row with different superscripts ^a,b^ differ significantly.

## Data Availability

The data presented in this study are available upon request from the corresponding author.
